# The Role of Adherence Thresholds for Development and Performance Aspects of a Prediction Model for Direct Oral Anticoagulation Adherence

**DOI:** 10.3389/fphar.2019.00113

**Published:** 2019-02-19

**Authors:** Carmen Ruff, Ludmila Koukalova, Walter E. Haefeli, Andreas D. Meid

**Affiliations:** Department of Clinical Pharmacology and Pharmacoepidemiology, University of Heidelberg, Heidelberg, Germany

**Keywords:** adherence, direct oral anticoagulants (DOACs), rivaroxaban, clinical prediction model, pharmacology/pharmacotherapy, claims data

## Abstract

Patients who do not sufficiently adhere to their dosing regimens will, ultimately, do not get the full benefit of their medication. For example, if direct oral anticoagulants (DOAC) are not taken continuously, an intervention to improve adherence or maintain persistence will show direct effects on clinical outcomes. Usually, adherent patients are defined by taking ≥80% of their medication. The resulting binary adherence status from this threshold can as well be used for predictive classification. Thus, the threshold can determine the prediction model’s performance to identify patients at risk for poor adherence by this binary adherence status. In this perspective, we propose a plan for model development and performance considering the threshold’s role. Concerning development demands, we extracted predictors from a systematic literature search on DOAC adherence to be used as a core set of candidate predictors. Independently, we investigated how well a future model would technically have to perform by modeling drug intake and thromboembolic events based on a rivaroxaban pharmacokinetic-pharmacodynamic model. Using this simulation framework for different thresholds, we projected the impact of an imperfectly predicted adherence status on the event risk, and how imperfect sensitivity and specificity affect the cost balance if a supporting intervention was offered to patients classified as non-adherent. Our simulation results suggest applying a rather high threshold (90%) for discrimination between patients at low or high risk for non-adherence by a prediction model in order to assure cost-efficient implementation.

## Introduction

Key advantages of direct oral anticoagulants (DOAC) are efficacy (at least as good as observed with warfarin), rare intracerebral bleeding events, and the absence of close monitoring requirements (Rivera-Caravaca et al., 2017). However, despite these benefits, a significant number of treated atrial fibrillation (AF) patients will experience (recurrent) stroke (Ruff et al., 2015; Shinoda et al., 2018). One of the most frequent causes of non-response is the patients’ failure to take their medicines as prescribed (non-adherence) (Steffel et al., 2018).

Each stage of medication adherence (initiation, implementation, discontinuation) as proposed by the ABC (Ascertaining Barriers to Compliance) project around Vrijens et al. (2012) plays an important role in the success of an intended drug therapy. In long-term DOAC therapies, roughly 40–60% of the patients are non-adherent with numbers increasing with the duration of treatment (Johnson et al., 2016; Brown et al., 2017; Collings et al., 2017; McHorney et al., 2017; Sorensen et al., 2017; Lip et al., 2018). This is of high clinical relevance because the risk for all-cause mortality and stroke increases by 13% per 10% decline in DOAC adherence (Shore et al., 2014).

An intuitive solution would be an intervention that improves poor adherence in eligible patients (Raparelli et al., 2017; Steffel et al., 2018). Such a supporting program is carried out best before the initiation of a long-term therapy, i.e., in parallel with starting anticoagulation. In this context, a key question is who should be enrolled in such an intervention program. In claims data, adherence is derived as a continuous measure (Dima and Dediu, 2017) to which a threshold is applied for classification into adherent and non-adherent patients [e.g., usually based on a proportion of days covered by medication (Brown et al., 2017; McHorney et al., 2017; Sorensen et al., 2017)]. It is noteworthy, though, that claims data do not represent the gold standard to measure adherence (Vrijens and Heidbuchel, 2015) and provide only information on the date of the refill so that measurement error has to be considered whenever interpreting a model developed from this data source. Identification of patients in need of adherence support will thus inherently rely on such a threshold as well. Surprisingly little is known about which threshold should be applied when developing a prediction model using claims data.

By the identification of patients in need we technically mean prediction of future DOAC adherence, covering implementation and persistence after initiation was successful according to the ABC taxonomy. The benefits of predicting anticoagulation quality have been impressively shown for vitamin-K antagonists (Apostolakis et al., 2013; Proietti and Lip, 2015). Nevertheless, there are many obstacles when predicting adherence from electronic routine data such as health insurance claims data (Steiner, 2010). Previous attempts have been criticized for their modest performance, which may be explained by the fact that easily measurable clinical attributes (e.g., diagnoses) rather than (surrogates for) individual behavior and beliefs were mainly used as candidate predictors. Hence, when developing a novel prediction model for DOAC adherence, it is quite obviously necessary in our opinion to consider derived variables (predictors) mimicking personal characteristics beyond usually explored factors such as age, sex, or comorbidities. For example, individual refill history of a patient is a strong predictor of future (non-)adherence behavior (van Onzenoort et al., 2011). Discontinued and re-started medications may also be informative, as well as various characteristics to be derived from them, such as swallowing deficits (Schiele et al., 2013) or the capability to cope with complex regimens (Witticke et al., 2013).

The aims of this study are thus to give a perspective of (1) which predictors should be considered when developing such a model predicting future DOAC adherence, and (2) how well this model would technically have to perform in order to be applied in a cost-efficient manner. Both goals explicitly deal with the (potential) impact of thresholds on model development (1) and model performance (2). To approach the first goal, factors commonly associated with DOAC non-adherence in regard to all processes defining adherence were identified in a systematic literature search. We then investigated the performance requirements for such a prediction model to be implemented in a cost-efficient manner. Claims data was considered as a promising future area of application of such a model.

## Materials and Methods

### Determination of Necessary Elements for Model Development (Candidate Predictors)

We conducted a literature search following the PRISMA statement (Moher et al., 2009) in the PubMed database with the aim of identifying predictor variables associated with adherence or persistence to DOACs in populations with AF. Accordingly, the details of the review protocol are summarized in the Supplementary Material 1. In brief, records were systematically identified by an extensive list of search terms including all possible adherence definitions according to the ABC taxonomy and all distinct concepts. The aim was to obtain a comprehensive set of candidate predictors through diligent and broad exploration across all adherence stages of the ABC taxonomy. Concerning study (original publication) selection, titles and abstracts were screened by two independent reviewers (CR and LK), while disagreements were solved by a third reviewer (ADM). Likewise, we screened the corresponding full-texts and extracted study characteristics, population characteristics, adherence measurement technique, thresholds for adherence (if applicable), results of study analyses in terms of associated predictor variables (either quantitatively or qualitatively) with (non-)adherence and (non-)persistence (discontinuation) as well as their validity (for a full list of criteria, see Supplementary Material 1). Thus, quantitative results of study analyses (either univariate or multivariate) were required to be significantly associated at the 0.05 level, while qualitative results of study analyses were included if the study assigned a clear link to DOAC adherence or persistence. Among excluded research articles without original data, we checked cited references in order to potentially include them as articles from other sources, to which the same assessment was applied. Records were analyzed in an explorative way according to their predictors (harmonized description) and the number of statistically significant results of study analyses for each candidate predictor with respect to the number of original publications was extracted.

The predictors that were finally identified in the categories of univariate, multivariate, or qualitative results of study analyses were grouped according to five dimensions of adherence (World Health Organization [WHO], 2003). To explore consistency between previous original publications, we extracted the direction of multivariate results of study analyses in the subset of eligible original publications. Continuous predictor variables reported as categories were interpreted on their original continuous scale assuming a linear effect across the categories.

### Determination of Requirements for Model Performance

In addition to identifying candidate predictors for model development, we aimed to investigate how well such a prediction model would technically have to perform in order to be of clinical benefit and of practical relevance in terms of cost-effectiveness. Therefore, we combined three elements describing (A) the clinical setting, (B) the prediction model setting, and (C) the implementation setting. To begin with the clinical setting (A), Modeling and Simulation (*M&S*) techniques were applied to investigate the impact of (non-)adherence and corresponding threshold effects on clinical outcomes, in particular thromboembolic events under AF treatment with the exemplary DOAC rivaroxaban. Of note, we modeled random non-adherence and did not consider non-persistence for reasons of simplicity. An extended pharmacokinetic-pharmacodynamic (PKPD) model yielded event rates in adherence groups separated at different thresholds as well as the risk for such an event by comparing these groups. Concerning the prediction model setting (B), we examined how the risk estimate between groups below and above a certain threshold is affected by imperfect discrimination between these groups. This relates to imperfect sensitivity and specificity. Considering the implementation of such a model to assess cost savings attributed to an intervention (C), we projected its cost-effective application. Based on an adherence improvement of some poorly adhering patients identified by the prediction model, we calculated cost balances by offsetting the interventional costs and saved costs by potentially averted clinical events. A detailed simulation protocol (Supplementary Material 2) summarizes the course of action including necessary assumptions. We briefly note that all assumptions were based on either established models (e.g., Girgis et al., 2014), empirically derived estimates (e.g., Kolominsky-Rabas et al., 2006; Connolly et al., 2009; Chapman et al., 2010; Patel et al., 2011), or clinically reasonable expectations.

## Results

### Perspective for Model Development: Candidate Predictors From the Literature

The PRISMA flowchart (see Supplementary Material 1) summarizes the systematic literature search with 54 identified studies (original publications) contributing to counted frequencies; among them, 26 studies provided multivariate analyses results. Overall 14 studies (58% of the total 24 studies dealing with non-adherence) defined non-adherence by a threshold to distinguish between adherent and non-adherent patients. The most common threshold was ≥80%, which was applied by 12 studies (86% of all studies using a threshold). One study compared patients with 100% adherence with patients being not fully adherent (Polymeris et al., 2016), while another study used three categories of low, medium, and high adherence (Manzoor et al., 2017).

Most studies exploring different thresholds used them primarily to report drug-specific adherence rates (Brown et al., 2016; Marquez-Contreras et al., 2016; Polymeris et al., 2016; Brown et al., 2017; McHorney et al., 2017). Only two studies investigated associations of predictor variables with non-adherence at different thresholds (Manzoor et al., 2017; Jacobs et al., 2018). In these publications, some predictors were inconsistently associated with non-adherence at the applied thresholds of 66–90, 80, and 90%, respectively.

**FIGURE 1:**
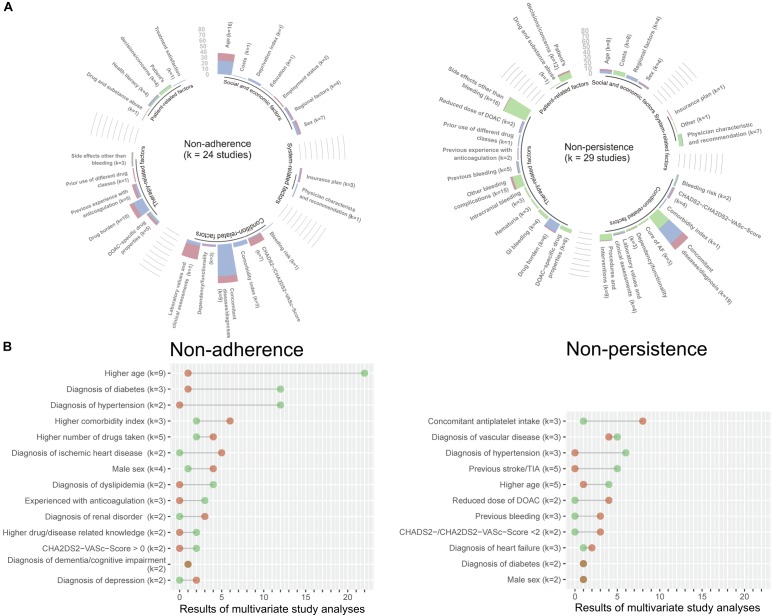
Results from the PubMed literature search on potential predictors of DOAC adherence and persistence. The analyses of the published data are coded in green (qualitative), blue (multivariate), and red (univariate). The height of the bar indicates the number of extracted statistically significant results for each predictor, where *k* denotes the number of original publications contributing to the respective predictor **(A)**. For multivariate estimates appearing in more than one original publication, the numbers and directions of the results from the subset of multivariate analyses are highlighted in a lollipop plot. Green dots indicate a negative association with non-adherence or non-persistence (protective constellation), while red dots show a positive association with non-adherence or non-persistence (risk constellation). The dots are connected by a straight line to visualize the diverging frequencies of results for each predictor; the longer the line, the clearer the conclusion for the predictor and the shorter the line the more conflicting the predictor. *K* denotes the number of original publications for each predictor **(B)**.

Regarding published candidate predictors, we identified age (16 studies) and drug burden (10 studies) as the two most frequent predictor variables that were statistically significantly associated with non-adherence, while co-morbidity (56 analyses) was most often identified in multivariate analyses (Figure 1A). Similarly, co-morbidity (19 studies) was among the most frequently statistically significant results for discontinuation (non-persistence). Further frequently associated results were bleeding-related events and other side effects. Multivariate results were largely consistent for both non-adherence and discontinuation (non-persistence) (Figure 1B).

### Perspective for Model Performance

**FIGURE 2:**
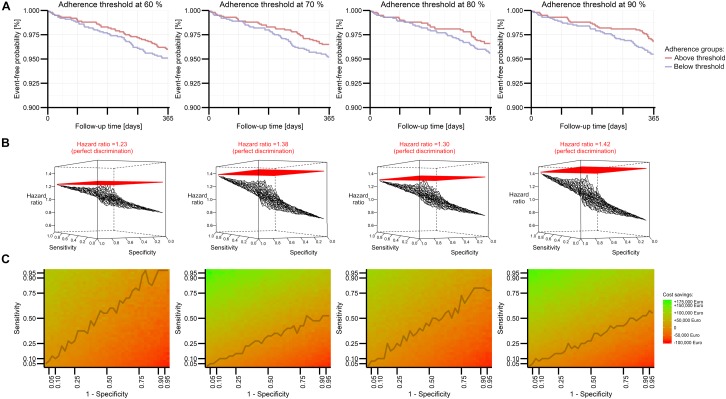
Perspective for model performance in predicting future DOAC adherence explored by Modeling and Simulation for four adherence thresholds. An extended rivaroxaban PKPD model (see Supplementary Material 2) describes the event-free probability of stroke or systemic embolism in terms of percentage adherence to administration regimen, while these groups are determined by several thresholds. Kaplan–Meier plots visualize the effect size of *one* simulation **(A)**. A three-dimensional mesh plot illustrates detectable effect sizes if the model imperfectly allocates patients to groups above and below a certain threshold in terms of sensitivity and specificity **(B)**. Expected cost savings from the model’s implementation are visualized in a heat plot based on expected costs and benefits for various performance estimates **(C)**. The line connects the smallest sensitivity values resulting in a positive cost balance.

Non-adherence patterns and attributed clinical risks were successfully incorporated into PKPD models to yield reasonable clinical effects (e.g., 4% yearly event rate in the simulated subsample at the 80% threshold, hazard ratio of 1.09 between subgroups of 70 and 80% adherence, respectively). By separating the population with various adherence rates at a certain threshold, we obtained groups with clearly distinguishable survival curves representing the event-free proportion (Figure 2A). Lowest effect sizes for the group comparison resulted from the 60% threshold, while the 90% threshold yielded the highest estimate (hazard ratio of 1.23 vs. 1.42). It is noteworthy that although the simulation input parameters assumed a strong clinical effect in the simulation framework, the sample size of 2,000 patients used for the simulation framework was not sufficiently large to reveal statistically significant results in the Cox model or to draw inferences from the Kaplan–Meier curves.

Based on these threshold-dependent adherence effects, we applied imperfect discrimination of the threshold-separated groups. This mimicked the performance of an intended prediction model. Imperfect assessment of the true group status at various levels of sensitivity and specificity markedly decreased the detectable effect size (Figure 2B). Despite the steady influence of both limited sensitivity and specificity, poor specificity appeared to have a greater impact than poor sensitivity. At an exemplary 90% threshold, a hazard ratio of 1.13 was obtained between imperfectly detected groups with 12.5% sensitivity and 87.5% specificity, whereas a hazard ratio of 1.02 obtained between imperfectly detected groups with 87.5% sensitivity and 12.5% specificity. The detectable effect size was related to the extrapolated cost savings of a potential intervention, which were indeed larger for the 90% threshold, if higher specificity was preferred. This corresponds to net cost balances which are driven rather by specificity than sensitivity, especially for the 90% threshold (Figure 2C). Of note, cost-effectiveness could be accomplished at any investigated threshold with reasonable discrimination performance.

## Discussion

Addressing the clinically relevant challenge of poor adherence to DOAC treatments, our study diligently explored contemporary evidence to propose a plan facilitating the development of a model predicting future DOAC adherence of a patient exemplified by rivaroxaban. For the first time, we are presenting a comprehensive plan including associated predictor variables as candidate predictors together with expected requirements and benefits when developing such a model in an AF population in the future. Considering the burden of disease and potential benefits, our results should help guiding future research when an adherence model is applied and followed by suitable interventions aiming to improve or maintain adherence to medications.

We found a considerable number of potentially associated predictors related to all different dimensions of DOAC adherence and persistence. Taking a closer look at the most frequently reported predictors (e.g., age, sex, and comorbidities), one has to admit that they are already well known, both for specific populations, particular drug classes, and for adherence in general (Kardas et al., 2013). Some predictors might be difficult to operationalize in claims data, though. Most of our identified studies used the common threshold of 80% to distinguish between adherent and non-adherent DOAC patients and it remains unclear whether this choice influences the (statistical) association of predictor variables. Current evidence is lacking for a choice of this threshold for DOACs; this choice appears rather justified by general conventions (Gellad et al., 2017) and therefore an appropriate threshold needs to be empirically investigated. Our simulation considered clinical and economic consequences of non-adherence and suggests that patients may benefit most if thresholds to separate adherent from non-adherent patients are set rather high. With higher thresholds, comparatively smaller sensitivity is needed than with lower thresholds, thus also facilitating the demands on model performance.

Our study has several limitations: our literature search was able to provide merely exploratory results due to the heterogeneous nature of the eligible publications. The studies ultimately included were heterogeneous, consisted of many different AF (sub-) populations characterized by different co-medications and different comorbidities. This non-uniform setting may have influenced the results. Further on, methodological approaches differed markedly ranging from data collection by questionnaires to automatic assessments from claims data. Inconsistencies were equally prominent concerning, for example, the measurement of adherence or the definition of diagnoses as predictor variables.

Another potential bias may be introduced by several results obtained from one study (population), i.e., all analyses ever conducted in a distinct study (original publication) were included. To account for this influence, we always give the total number of studies for each predictor found. Needless to say, our recommendations do not guarantee the successful development of the intended prediction model, although our simulation results suggest excellent prospects for the example drug rivaroxaban. Due to drug-related aspects (e.g., dosing regimens, safety profiles), such a model would have to make predictions for each DOAC separately. Setting the unknown *M&S* parameters, we chose clinically reasonable estimates and thresholds at a pragmatic sample size achievable for most health insurance companies: considering the incidence of AF in Germany [4.112/1,000 person-years (Wilke et al., 2013)], a health insurance company would need about half a million eligible health-insured patients. Studying imperfect sensitivity and specificity in order to extrapolate the demands of a prediction model for future DOAC adherence, we assumed that other sources of bias were absent, first of all that adherence (in terms of implementation and discontinuation) can be measured without error. This has to be considered whenever interpreting a model predicting future (non-)adherence, because this bias goes beyond prediction error (and is present in claims data). We therefore expect effects to be somewhat smaller, but at the same time we emphasize the need for a clinical study examining (and confirming) these effects. In our simulation framework, we had no precise information pertaining to the distribution of adherence percentages, the severity of disease, or the interventions’ effectiveness and costs, for all of which we had to make guesses. Nevertheless, it is possible to update our simulation framework with empiric estimates or real input values for intervention costs. This leads *vice versa* to another potential application, namely one that enables us to study how effective and how expensive an intervention would have to be when a prediction model with distinct sensitivity and specificity has already been developed. Moreover, future studies could address further pharmacoeconomic analyses (e.g., opportunity costs considering averted events induced by optimal adherence).

In conclusion, adherence and its associated predictor variables are extensively explored in the literature, while their suitability as predictors of future DOAC adherence needs to be empirically confirmed by actually developing the outlined prediction model. Beside these candidate predictors, patient characteristics, behavior, or attitudes going beyond these ubiquitous and readily available predictor variables should be explored. In order to assure cost-efficient implementation, a higher adherence threshold appears advisable to allocate interventional resources to patients at risk of falling below the 90% threshold. Clinical utility should focus on specificity to ensure the highest achievable benefit from such an intervention.

## Data Availability

Data extraction forms from the literature search and simulated data sets are available upon request from the corresponding author.

## Author Contributions

ADM, CR, and LK were involved in preparation of the review protocol and development of the search strategy. CR and LK were involved in assessing abstracts and all full-text copies. All authors were responsible for data synthesis (where ADM acted as the third person for consultancy in cases of disagreement between two reviewers). ADM conducted the analysis related to the demands for model performance. ADM, CR, and WEH drafted and revised the manuscript. All authors read and approved the final manuscript.

## Conflict of Interest Statement

WH received consulting honoraria, speaker fees, or travel support outside the submitted work from the following companies: Bristol-Myers Squibb, Boehringer Ingelheim, Bayer HealthCare, Daiichi Sankyo, and Pfizer. The remaining authors declare that the research was conducted in the absence of any commercial or financial relationships that could be construed as a potential conflict of interest.
