# Supplementary material for: The Role of Adherence Thresholds for Development and Performance Aspects of a Prediction Model for Direct Oral Anticoagulation Adherence
Source: Front Pharmacol. 2019 Feb 19;10:113. doi: 10.3389/fphar.2019.00113 (PMC6389873; doi:10.3389/fphar.2019.00113)
Supplement: Supplementary file 1 [file Data_Sheet_1.pdf]

Electronic Supplementary Material to

The Role of Thresholds for Development and Performance Aspects of a Prediction Model for Direct Oral Anticoagulation Adherence

Ruff *et al.*

Frontiers in Pharmacology

Review Protocol to "The Role of Thresholds for Development and Performance Aspects of a Prediction Model for Direct Oral Anticoagulation Adherence"

Carmen Ruff[1,a], Ludmila Koukalova[1,a], Walter E. Haefeli[1,b*] and Andreas D. Meid[1,b]

[1]Department of Clinical Pharmacology and Pharmacoepidemiology, University of Heidelberg, Im Neuenheimer Feld 410, 69120 Heidelberg, Germany

[a,b] equal contribution, respectively

Correspondence*:

Professor Walter E. Haefeli, Department of Clinical Pharmacology and Pharmacoepidemiology, University of Heidelberg, Im Neuenheimer Feld 410, 69120 Heidelberg, Phone: +49 6221 56 8740, Fax: +49 6221 56 4642, E-Mail: walter.emil.haefeli@med.uni-heidelberg.de.

Contributions of the protocol authors:

WEH is the guarantor of this review. ADM, CR, and LK were involved in the preparation of the review protocol, development of the search strategy, development of inclusion and exclusion criteria. CR and LK were involved in assessing abstracts and all full-text copies. All authors were responsible for data synthesis (where ADM acted as the third person for consultancy in disagreements between two reviewers).

Sources of financial or other support for the review/review protocol:

ADM and CR were supported by grants 01VSF16012 and 01VSF18019 from the German Innovation Fonds and 01GY1320B from the German Ministry of Education and Research (BMBF). The funders had no role in the design, decision to publish, or preparation of the review protocol.

1. The background to the review (briefly)

Medication nonadherence is a common problem hindering the effectiveness of pharmacotherapy. The most striking impact can be seen in conditions, where drug underuse quickly results in unwanted adverse events. One of these conditions is atrial fibrillation, in which anticoagulation treatment, represented mainly by oral anticoagulants, plays an important role. Patients starting anticoagulation therapy might thus profit from an adherence intervention intended for the potential thromboembolic event prevention. In order to determine, which patient might be non-adherent in the future, a prediction model can be created by using information about former adherence and certain factors influencing adherence in atrial fibrillation subjects.

2. The objectives or purpose of the review

The aim of this review is to identify clinical factors of medication non-adherence or discontinuation in atrial fibrillation patients, treated with direct oral anticoagulants (DOACs).

3. The inclusion and exclusion criteria for considering the studies in the review

What type of studies?

- All kinds of articles, except for editorials, study protocols and meeting/conference abstracts
- Any type of study design (including quantitative and qualitative research, such as case reports e.g.)

Types of participants

- Adult patients (≥ 18 years) with atrial fibrillation receiving an anticoagulation pharmacotherapy (DOACs)

Types of intervention

- NA

Comparator

  - if study design allows: adherent vs. non-adherent group, persistent vs. non-persistent/discontinued group

Outcome

- A measure of (non)adherence or discontinuation (mandatory) and possibly associated clinical outcomes (e.g., stroke, bleeding, among others) in addition (optionally)

Inclusion:

- Articles describing drug-level or patient-level variables associated with or predictive for non-adherence (incl. discontinuation) to direct oral anticoagulants (if results are mixed, to all oral anticoagulants)
- Articles about patient populations, where not all patients suffer from (persistent) atrial fibrillation, are also to be included, if the main focus of the paper matches the first inclusion criterion
- Articles dealing with drug discontinuation (non-persistence) are to be considered, too
- Relevant articles should address underuse as insufficient intake of direct oral anticoagulants
- Any adherence measurement technique is of importance (self-reported adherence, claims-derived adherence, among others)
- Articles with different thresholds and articles with no thresholds for adherence (threshold for non-adherence may vary among studies, but all articles are eligible to be included in the first place – if other inclusion criteria are fulfilled)
- Any ambulatory setting is of interest
- Any setting indicating that the patients themselves are responsible for their medication intake/administration/organization
- Articles with a publication date after the approval of DOACs for atrial fibrillation (i.e. FDA approval date for first DOAC for atrial fibrillation - Dabigatran: October 2010)
- Articles only indicating an effect estimate for total adherence in populations taking many drugs, administering DOACs and/or other oral and non-oral anticoagulants, respectively (if effect estimate is not separately indicated for atrial fibrillation population with oral anticoagulants)
- Articles indicating any possible associated factor with (non-)adherence (incl. (non-)persistence or discontinuation) in DOACs
- Articles indicating any possible associated factors for (non-)adherence (incl. (non-)persistence or discontinuation) in DOACs AND Vitamin K-antagonists (if results for DOACS are separately indicated; if results for DOACs AND Vitamin K are presented as a compilation of factors, not differentiating between the factors for DOACs OR for Vitamin K)
- The inclusion period of the study must be applicable to the time period of the market authorization for DOACs

Exclusion:

- Articles obviously lacking drug-level or patient-level variables associated with non-adherence or predictive for non-adherence (incl. discontinuation) to direct oral anticoagulants (e.g. articles describing the relationship of poor adherence with adverse outcomes alone, or articles providing only information about non-adherence rates without investigating their causes or only contemplating the causes)
- Articles exclusively addressing drug overuse as overdosing
- Articles focusing only on primary non-adherence (i. e. prescription filling)
- Compliance with guidelines and thus decisions for treatment or non-treatment (underuse as prescribing omissions)

- Non-adherence to regimens such as diet, physical activity, etc.; these articles can be included only if they contain separate measurements of adherence to DOACs
- Studies with inpatients only
- Studies with patients in nursing homes etc. (where one would expect that patients do not take care of the medication themselves)
- Studies with underage patients, studies indicating that parents or other responsible persons care about administering the drugs
- Articles in other languages than English, German, French, Czech, Spanish, Italian and Slovak
- Author´s opinions in reviews, comments, etc.; these articles are only used as an additional source for primary research studies Noprimary research articles; these articles are only used as an additional source for primary research studies
- Articles providing reasons for discontinuation or non-adherence (qualitative results) without a clear link to DOAC involvement
- Interventional studies, comparing adherence rates before and after the intervention studies giving only adherence rates as an outcome or comparing different adherence rates for specific drugs

4. Information sources

- electronic databases: PubMed
- if needed authors were approached for appendix/supplemental material

5. The search strategy

- electronic database: PubMed (https://www.ncbi.nlm.nih.gov/pubmed/)
- No language restriction
- Found articles are sorted by ”Most recent”
- Search term was entered as follows:**("Prognosis"[Mesh] OR "impact"[ALL] OR "influence"[ALL] OR "affect"[ALL] OR "factor"[ALL] OR "factors"[ALL] OR "reason"[ALL] OR "reasons"[ALL] OR "predict"[ALL] OR "determinant"[ALL] OR "determinants"[ALL] OR "risk"[ALL] OR "risks"[ALL] OR "causes"[ALL] OR "hazards models"[ALL] OR "correlation"[ALL] OR "correlate"[ALL]) AND ("Atrial Fibrillation"[Mesh] OR "atrial fibrillation"[TIAB]) AND ("Medication Adherence"[Mesh] OR "adherence"[TIAB] OR "non-adherence"[TIAB] OR "nonadherence"[TIAB] OR "compliance"[TIAB] OR "non-compliance"[TIAB] OR "noncompliance"[TIAB] OR "persistence"[TIAB] OR "discontinue"[TIAB] OR "non-persistence"[TIAB] OR "nonpersistence"[TIAB] OR "discontinuation"[TIAB] OR "discontinuing"[TIAB] OR "adherent"[TIAB] OR "compliant"[TIAB] OR "persistent"[TIAB]) AND ("oral anticoagulant"[ALL] OR "direct oral anticoagulant"[ALL] OR "oral anticoagulants"[ALL] OR "direct oral anticoagulants"[ALL] OR "non-VKA"[ALL] OR "non-vitamin K antagonist"[ALL] OR "OAC"[ALL] OR "OACs"[ALL] OR "DOAC"[ALL] OR "DOACs"[ALL] OR "NOAC"[ALL] OR "NOACs"[ALL] OR "dabigatran"[ALL] OR "rivaroxaban"[ALL] OR "apixaban"[ALL] OR "edoxaban"[ALL] OR "rivaroxaban"[ALL] OR "dabigatran"[ALL] OR "direct thrombin inhibitor"[ALL] OR "direct thrombin inhibitors"[ALL] OR "Factor Xa Inhibitors"[Mesh] OR "direct**

factor Xa inhibitor"[ALL] OR "direct factor Xa inhibitors"[ALL] OR "oral factor Xa inhibitor"[ALL] OR "oral factor Xa inhibitors"[ALL] OR "new oral anticoagulant"[ALL] OR "new oral anticoagulants"[ALL]) Filters: **Publication date from 2010/01/01**

- records identified through other sources, e.g. reference lists

6. Identification of how the quality of the articles will be assessed

1) Screening all articles provided by the specified search, which includes reading the title, abstract; entering results in separate excel sheets (Excel software, Office 2010); done by two independent reviewers (CR, LK)
2) Comparison of screening results using R software version 3.4.4
3) In case of disagreement of the two reviewers, a third reviewer (ADM) decides if the article is eligible for full-text reading
4) In the second phase, reading full article and assessment against the inclusion/exclusion criteria; CR, LK enter independently the data into the data extraction form (see below). The other reviewer screens the respective data extraction form (word document; word software Office 2010) and included further results, if necessary.
5) In case of disagreement between the two reviewers, a third reviewer (ADM) decides if the article is eligible for inclusion
6) Risk of bias of individual studies was not assessed due to vast heterogeneity of the observational and exploratory results

7. Data extraction

- a patient-level or drug-level variable to be found either predictive (i.e. included in the finally reported prediction model) or associated with (non)adherence to oral anticoagulants (i.e. achieving a p-value of smaller 0.05 in the multivariate model or univariate comparison, if no multivariate model exists → for quantitative studies)

Table 1 - Template of the Data Extraction Form (DEF) with explanations (in grey)

Reviewer	Reviewer´s initials
Bibliographic details of the study	Vancouver style citation
Publication type	☐ Review, Meta-analysis<br>☐ Original research<br>☐ Other (indicate type, e. g. guideline, comment)

Research methods	☐ Qualitative research (frequencies)<br><br>☐ Quantitative research<br><br>☐ Other (expert opinion, consensus (doctor´s opinion), Delphi procedures, …)
Decision for inclusion	☐Yes ☐No
Reasons for exclusion	
**Below only for included articles**	
Purpose of the study	Brief description of the main aim, with which the study was carried out
Study design	Strategy used for the solution of the research problem (e. g. prospective, randomized, multicenter)
Data sources	e.g., secondary/routine data (claims data, registries, …) or primary data
Population (sample)	Description of participants<br><br>- age:<br><br>- % of female participants:<br><br>- size of the sample:<br><br>- % of patients with atrial fibrillation<br><br>- type of atrial fibrillation:<br><br>☐ paroxysmal (%) ☐ persistent (%) ☐ permanent (%)<br><br>- previous stroke: (%)<br><br>- drugs under investigation:<br><br>- treatment naïve: (%)<br><br>- DOAC-naïve: (%)
Follow-up time	e.g., 12 months
Adherence measurement technique	Means used to assess medication adherence (e. g. survey/questionnaire, MEMS, insurance claims data, pill count,

	direct observation)
Outcome measures	☐ Proportion of days covered (PDC)<br><br>☐ Medication possession ratio<br><br>☐ Treatment discontinuation<br><br>☐ Other (e. g. level of drug in the blood, biological marker, assessment of the patient's clinical response)
Results	Identified factors influencing adherence, please add in brackets to all factors a plus sign, if they promote adherence, a minus sign for factors promoting non-adherence
Results validity	In case of quantitative results, univariate or multivariate result<br><br>In case of qualitative results, percentage frequency of all patients<br><br>In case of opinion, ideas were compiled by:<br><br>☐One author ☐Several authors ☐Expert panel
Was threshold used for differentiation of adherent and nonadherent group? If yes, what numerical value was used?	☐Yes - Threshold percentage<br><br>☐No
Notes	

8. PRISMA flowchart

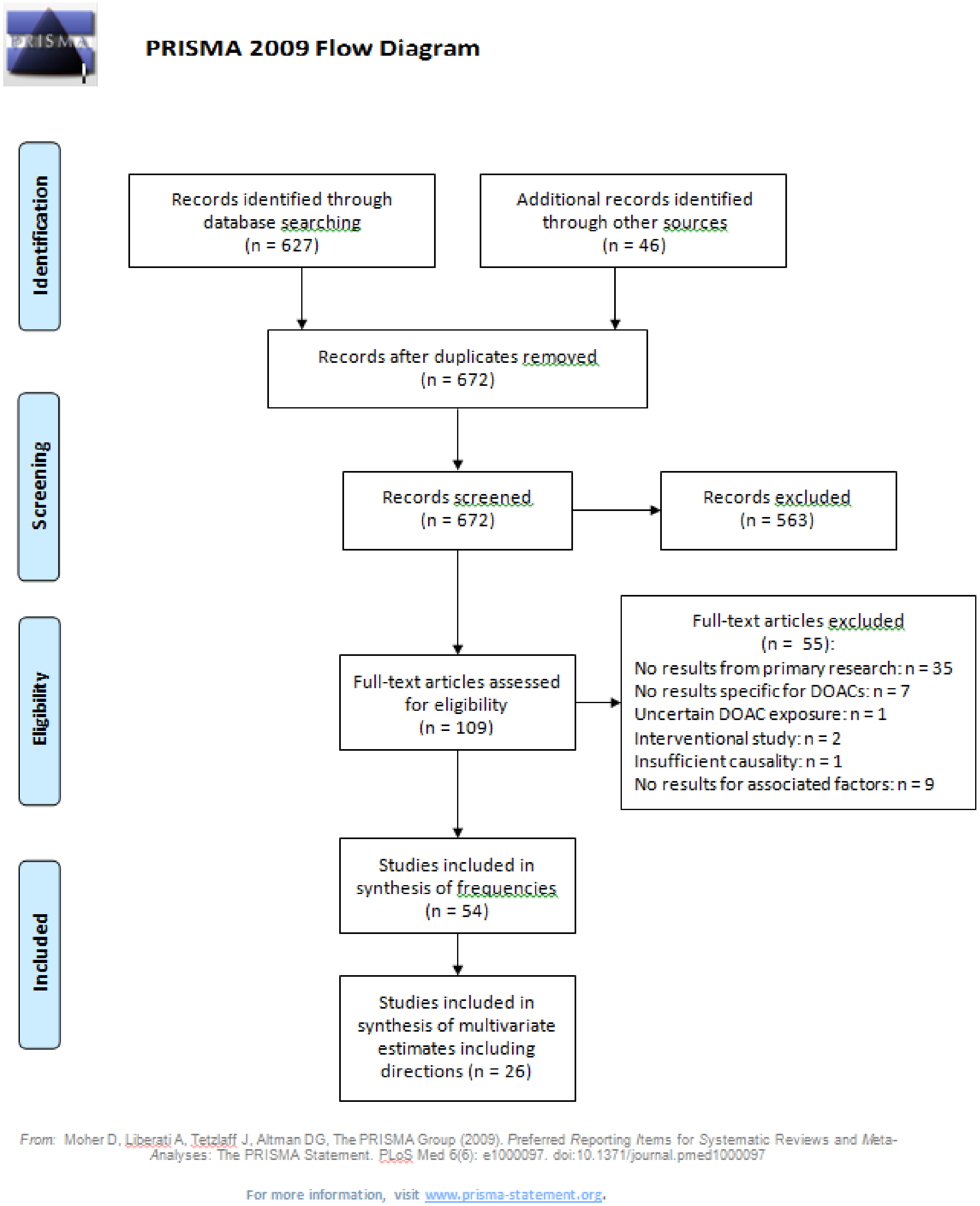

Figure 1 - PRISMA Flow Chart

9. Exclusion of factors obtained by multivariate analysis
   a. From all the factors obtained by the literature search, we exclude those regarding the frequency of drug administration (i.e. once- or twice-daily administration)because the dosing regimen is a non-changeable factor directly attached to the corresponding drug and its market approval for a particular indication
   b. results referring to adherence for a particular drug or rather comparison of different adherence rates for particular drugs (e.g. adherence rate for Rivaroxaban is different to adherence rate for Dabigatran) will not be reported, because the decision of the selection of a particular drug for a patient is done by a physician and depends on many different considerations about the patients' status, comorbidities and behavior.
   c. To ensure a world-wide applicability of our prediction model, we excluded those, that referred to the patient´s residence (e.g. residence in the West of the USA)
   d. overseas department category as a deprivation index category will not be reported but excluded as a regional factor, because this category cannot be generally estimated as a more deprived area
   e. factors referring to the insurance type will not be reported because of many differences in the worldwide healthcare systems
   f. Further on, it must be noted that continuous variables reported as categories in the original publication were assumed to show a linear effect over the categories. If the direction of effects were not consistent across these categories, though, no result was extracted for this variable.

10. Synthesizing the evidence:
   a. Data were not synthesized in a meta-analytic manner, but only frequencies of associated factors were extracted.
   b. Assessment of meta-bias was not carried out, because we did not apply a meta-analytic approach and our objectives are purely exploratory.

11. Type of summary planned:
   a. all factors obtained by the literature search (providing a statistically significant result for quantitative factors) will be reported, using a circular stacked barplot: Colors would show the number of univariate, multivariate and qualitative results, whereas a set of factors between the gaps reflects one of the five adherence dimensions (e. g. patient-related), respectively. For instance, yellow would indicate the number of univariate, green multivariate and violet qualitative results.
   b. In order to give a convenient figure with appropriate results, factors will be summarized if possible and applicable (e.g. factor "age category 65-74 years" will be included in the summarized factor "age" in this figure)
   c. Multivariate results will be shown in a lollipop plot with 2 values, indicating the direction of the factor associated with adherence/persistence or non-adherence/non-persistence/discontinuation, respectively. Each factor would be shown in this graph by a red and a green dot, connected with a line. The position of a red dot would indicate the

number of analysis positively associated with nonadherence (i. e. risk factor), the position of a green dot the number of analysis negatively associated with non-adherence (i.e. protective factor). Therefore, the shorter the line between the red and the green dot, the more conflicting the factor.

d. In order to give a convenient figure with appropriate results, factors obtained by multivariate results will be summarized (e.g. factor "age category 65-74 years vs. ≤ 64 years" will be included in the summarized factor "higher age" in this figure). (for a comprehensive list of summarized factors, please see table 3)

RESULTS

12. Bibliographical data of included studies:

a. Articles identified via PubMed Search:

1. Horstmann S, Rizos T, Saribas M, Efthymiou E, Rauch G, Veltkamp R. Cognitive impairment is not a predictor of failure to adhere to anticoagulation of stroke patients with atrial fibrillation. Cerebrovasc Dis 2015;39:325-31.
2. Emren SV, Zoghi M, Berilgen R, Özdemir İH, Çelik O, Çetin N, Enhoş A, Köseoğlu C, Akyüz A, Doğan V, Levent F, Dereli Y, Doğan T, Başaran Ö, Karaca I, Karaca Ö, Otlu YÖ, Özmen Ç, Coşar S, Sümerkan M, Gürsul E, İnci S, Onrat E, Ergene O. Safety of once- or twice-daily dosing of non-vitamin K antagonist oral anticoagulants (NOACs) in patients with nonvalvular atrial fibrillation: A NOAC-TR study. Bosn J Basic Med Sci 2018;18:185-190.
3. Rolls CA, Obamiro KO, Chalmers L, Bereznicki LRE. The relationship between knowledge, health literacy, and adherence among patients taking oral anticoagulants for stroke thromboprophylaxis in atrial fibrillation. Cardiovasc Ther 2017;35.
4. Márquez-Contreras E, Martell-Carlos N, Gil-Guillén V, De La Figuera-Von Wichmann M, Sanchez-López E, Márquez-Rivero S, Gil-Gil I, Hermida-Campa E. Therapeutic compliance with rivaroxaban in preventing stroke in patients with non-valvular atrial fibrillation: CUMRIVAFA study. Curr Med Res Opin 2016;32:2013-2020.
5. Andrade JG, Krahn AD, Skanes AC, Purdham D, Ciaccia A, Connors S. Values and Preferences of Physicians and Patients With Nonvalvular Atrial Fibrillation Who Receive Oral Anticoagulation Therapy for Stroke Prevention. Can J Cardiol 2016;32:747-53.
6. Al-Khalili F, Lindström C, Benson L. The safety and persistence of non-vitamin-K-antagonist oral anticoagulants in atrial fibrillation patients treated in a well structured atrial fibrillation clinic. Curr Med Res Opin 2016;32:779-85.
7. Verdecchia P, Molini G, Bartolini C, De Filippo V, Valecchi F, Martone S, Aita A, di Giacomo L, Angeli F, Reboldi G. Safety of dabigatran in an elderly population: single center experience in Italy. Curr Drug Saf 2015;10:165-9.

8. Zalesak M, Siu K, Francis K, Yu C, Alvrtsyan H, Rao Y, Walker D, Sander S, Miyasato G, Matchar D, Sanchez H. Higher persistence in newly diagnosed nonvalvular atrial fibrillation patients treated with dabigatran versus warfarin. Circ Cardiovasc Qual Outcomes 2013;6:567-74.
9. Ho JC, Chang AM, Yan BP, Yu CM, Lam YY, Lee VW. Dabigatran compared with warfarin for stroke prevention with atrial fibrillation: experience in Hong Kong. Clin Cardiol 2012;35:E40-5.
10. Harper P, Pollock D, Stephens M. Dabigatran persistence and adherence in New Zealand: a nationwide retrospective observational study. BMJ Open 2018;8:e020212.
11. Collings SL, Vannier-Moreau V, Johnson ME, Stynes G, Lefèvre C, Maguire A, Asmar J, Bizouard G, Duhot D, Mouquet F, Fauchier L. Initiation and continuation of oral anticoagulant prescriptions for stroke prevention in non-valvular atrial fibrillation: A cohort study in primary care in France. Arch Cardiovasc Dis 2018;111:370-379.
12. Laube ES, Yu A, Gupta D, Miao Y, Samedy P, Wills J, Harnicar S, Soff GA, Mantha S. Rivaroxaban for Stroke Prevention in Patients With Nonvalvular Atrial Fibrillation and Active Cancer. Am J Cardiol 2017;120:213-217.
13. Königsbrügge O, Simon A, Domanovits H, Pabinger I, Ay C. Thromboembolic events, bleeding, and drug discontinuation in patients with atrial fibrillation on anticoagulation: a prospective hospital-based registry. BMC Cardiovasc Disord 2016;16:254.
14. Hellfritzsch M, Husted SE, Grove EL, Rasmussen L, Poulsen BK, Johnsen SP, Hallas J, Pottegård A. Treatment Changes among Users of Non-Vitamin K Antagonist Oral Anticoagulants in Atrial Fibrillation. Basic Clin Pharmacol Toxicol 2017;120:187-194.
15. Johnson ME, Lefèvre C, Collings SL, Evans D, Kloss S, Ridha E, Maguire A. Early real-world evidence of persistence on oral anticoagulants for stroke prevention in non-valvular atrial fibrillation: a cohort study in UK primary care. BMJ Open 2016;6:e011471.
16. Kachroo S, Hamilton M, Liu X, Pan X, Brixner D, Marrouche N, Biskupiak J. Oral anticoagulant discontinuation in patients with nonvalvular atrial fibrillation. Am J Manag Care 2016;22:e1-8.
17. Polymeris AA, Traenka C, Hert L, Seiffge DJ, Peters N, De Marchis GM, Bonati LH, Lyrer PA, Engelter ST. Frequency and Determinants of Adherence to Oral Anticoagulants in Stroke Patients with Atrial Fibrillation in Clinical Practice. Eur Neurol 2016;76:187-193.
18. Forslund T, Wettermark B, Hjemdahl P. Comparison of treatment persistence with different oral anticoagulants in patients with atrial fibrillation. Eur J Clin Pharmacol 2016;72:329-38.
19. Jacobs MS, Schouten JF, de Boer PT, Hoffmann M, Levin LÅ, Postma MJ. Secondary adherence to non-vitamin-K antagonist oral anticoagulants in patients with atrial fibrillation in Sweden and the Netherlands. Curr Med Res Opin 2018; (in press)
20. Deshpande CG, Kogut S, Laforge R, Willey C. Impact of medication adherence on risk of ischemic stroke, major bleeding and deep vein thrombosis in atrial fibrillation patients using novel oral anticoagulants. Curr Med Res Opin 2018;34:1285-1292.
21. Beyer-Westendorf J, Förster K, Ebertz F, Gelbricht V, Schreier T, Göbelt M, Michalski F, Endig H, Sahin K, Tittl L, Weiss N. Drug persistence with rivaroxaban therapy in atrial fibrillation patients-results from the Dresden non-interventional oral anticoagulation registry. Europace 2015;17:530-8.

22. Brown JD, Shewale AR, Talbert JC. Adherence to Rivaroxaban, Dabigatran, and Apixaban for Stroke Prevention for Newly Diagnosed and Treatment-Naive Atrial Fibrillation Patients: An Update Using 2013-2014 Data. J Manag Care Spec Pharm 2017;23:958-967.
23. Brown JD, Shewale AR, Talbert JC. Adherence to Rivaroxaban, Dabigatran, and Apixaban for Stroke Prevention in Incident, Treatment-Naïve Nonvalvular Atrial Fibrillation. J Manag Care Spec Pharm 2016;22:1319-1329.
24. Hu YF, Liao JN, Chern CM, Weng CH, Lin YJ, Chang SL, Wu CH, Sung SH, Wang KL, Lu TM, Chao TF, Lo LW, Chung FP, Hsu LC, Chen SA. Identification and management of noncompliance in atrial fibrillation patients receiving dabigatran: the role of a drug monitor. Pacing Clin Electrophysiol 2015;38:465-71.
25. Obamiro KO, Chalmers L, Lee K, Bereznicki BJ, Bereznicki LR. Adherence to Oral Anticoagulants in Atrial Fibrillation: An Australian Survey. J Cardiovasc Pharmacol Ther 2018;23:337-343.
26. Paquette M, Riou França L, Teutsch C, Diener HC, Lu S, Dubner SJ, Ma CS, Rothman KJ, Zint K, Halperin JL, Huisman MV, Lip GYH, Nieuwlaat R. Persistence With Dabigatran Therapy at 2 Years in Patients With Atrial Fibrillation. J Am Coll Cardiol 2017;70:1573-1583.
27. Emren SV, Şenöz O, Bilgin M, Beton O, Aslan A, Taşkin U, Açiksari G, Asarcikli LD, Çakir H, Bekar L, Bolat İ, Yayla Ç, Çelebi B, Dalgiç O, Çelik O, Şafak Ö, Akyel S, Güngör H, Düzel B, Zoghi M. Drug Adherence in Patients With Nonvalvular Atrial Fibrillation Taking Non-Vitamin K Antagonist Oral Anticoagulants in Turkey: NOAC-TR. Clin Appl Thromb Hemost 2018;24:525-531.
28. Jankowska-Polańska B, Katarzyna L, Lidia A, Joanna J, Dudek K, Izabella U. Cognitive function and adherence to anticoagulation treatment in patients with atrial fibrillation. J Geriatr Cardiol 2016;13:559-65.
29. Beshir SA, Chee KH, Lo YL. Factors associated with abrupt discontinuation of dabigatran therapy in patients with atrial fibrillation in Malaysia. Int J Clin Pharm 2016;38:1182-90.
30. Sauer R, Sauer EM, Bobinger T, Blinzler C, Huttner HB, Schwab S, Köhrmann M. Adherence to oral anticoagulation in secondary stroke prevention--the first year of direct oral anticoagulants. J Stroke Cerebrovasc Dis 2015;24:78-82.
31. Thorne K, Jayathissa S, Dee S, Briggs N, Taylor J, Reid S, De Silva K, Dean J. Adherence and outcomes of patients prescribed dabigatran (Pradaxa) in routine clinical practice. Intern Med J 2014;44:261-5.
32. Vedovati MC, Verdecchia P, Giustozzi M, Molini G, Conti S, Pierpaoli L, Valecchi F, Aita A, Agnelli G, Becattini C. Permanent discontinuation of non vitamin K oral anticoagulants in real life patients with non-valvular atrial fibrillation. Int J Cardiol 2017;236:363-369.
33. Suzuki T, Shiga T, Omori H, Tatsumi F, Nishimura K, Hagiwara N. Adherence to medication and characteristics of Japanese patients with non-valvular atrial fibrillation. J Cardiol 2017;70:238-243.
34. Beyer-Westendorf J, Ehlken B, Evers T. Real-world persistence and adherence to oral anticoagulation for stroke risk reduction in patients with atrial fibrillation. Europace 2016;18:1150-7.
35. Shiga T, Naganuma M, Nagao T, Maruyama K, Suzuki A, Murasaki K, Hagiwara N. Persistence of non-vitamin K antagonist oral anticoagulant use in Japanese patients with atrial fibrillation: A single-center observational study. J Arrhythm 2015;31:339-44.

36. Castellucci LA, Shaw J, van der Salm K, Erkens P, Le Gal G, Petrcich W, Carrier M. Self-reported adherence to anticoagulation and its determinants using the Morisky medication adherence scale. Thromb Res 2015;136:727-31.
37. Saliba L, Mondoly P, Duparc A, Bura-Rivière A, Maury P, Calmels V, Sallerin B, Pathak A, Montastruc JL, Bagheri H. [Factors Associated with Direct Oral Anticoagulants versus Vitamin K Antagonists in Patients with Non-valvular Atrial Fibrillation]. Therapie 2015;70:485-92.
38. Beyer-Westendorf J, Ebertz F, Förster K, Gelbricht V, Michalski F, Köhler C, Werth S, Endig H, Pannach S, Tittl L, Sahin K, Daschkow K, Weiss N. Effectiveness and safety of dabigatran therapy in daily-care patients with atrial fibrillation. Results from the Dresden NOAC Registry. Thromb Haemost 2015;113:1247-57.
39. Tsivgoulis G, Krogias C, Sands KA, Sharma VK, Katsanos AH, Vadikolias K, Papageorgiou SG, Heliopoulos I, Shiue H, Mitsoglou A, Liantinioti C, Athanasiadis D, Giannopoulos S, Piperidou C, Voumvourakis K, Alexandrov AV. Dabigatran etexilate for secondary stroke prevention: the first year experience from a multicenter short-term registry. Ther Adv Neurol Disord 2014;7:155-61.
40. Ho MH, Ho CW, Cheung E, Chan PH, Hai JJ, Chan KH, Chan EW, Leung GK, Tse HF, Siu CW. Continuation of dabigatran therapy in "real-world" practice in Hong Kong. PLoS One 2014;9:e101245.
41. Vanga SR, Satti SR, Williams J, Weintraub W, Doorey A. Discontinuation of oral anticoagulation preceding acute ischemic stroke--prevalence and outcomes: Comprehensive chart review. Postgrad Med 2015;127:791-5.
42. Douros A, Renoux C, Coulombe J, Suissa S. Patterns of long-term use of non-vitamin K antagonist oral anticoagulants for non-valvular atrial fibrillation: Quebec observational study. Pharmacoepidemiol Drug Saf 2017;26:1546-1554.
43. Manzoor BS, Lee TA, Sharp LK, Walton SM, Galanter WL, Nutescu EA. Real-World Adherence and Persistence with Direct Oral Anticoagulants in Adults with Atrial Fibrillation. Pharmacotherapy 2017;37:1221-1230.
44. Maura G, Pariente A, Alla F, Billionnet C. Adherence with direct oral anticoagulants in nonvalvular atrial fibrillation new users and associated factors: a French nationwide cohort study. Pharmacoepidemiol Drug Saf 2017;26:1367-1377.
45. Pisters R, van Vugt SPG, Brouwer MA, Elvan A, Ten Holt WL, Zwart PAG, Kirchhof P, Crijns HJGM, Hemels MEW. Real-life use of Rivaroxaban in the Netherlands: data from the Xarelto for Prevention of Stroke in Patients with Atrial Fibrillation (XANTUS) registry. Neth Heart J 2017;25:551-558.
46. Gorst-Rasmussen A, Skjøth F, Larsen TB, Rasmussen LH, Lip GY, Lane DA. Dabigatran adherence in atrial fibrillation patients during the first year after diagnosis: a nationwide cohort study. J Thromb Haemost 2015;13:495-504.
47. Luger S, Hohmann C, Niemann D, Kraft P, Gunreben I, Neumann-Haefelin T, Kleinschnitz C, Steinmetz H, Foerch C, Pfeilschifter W. Adherence to oral anticoagulant therapy in secondary stroke prevention - impact of the novel oral anticoagulants. Patient Prefer Adherence 2015;9:1695-705.

b. Additional articles identified by other sources:

1. McHorney CA, Ashton V, Laliberté F, Germain G, Wynant W, Crivera C, Schein JR, Lefebvre P, Peterson ED. Adherence to Rivaroxaban Compared with Other Oral Anticoagulant Agents Among Patients with Nonvalvular Atrial Fibrillation. J Manag Care Spec Pharm 2017;23:980-988.
2. Yao X, Abraham NS, Alexander GC, Crown W, Montori VM, Sangaralingham LR, Gersh BJ, Shah ND, Noseworthy PA. Effect of Adherence to Oral Anticoagulants on Risk of Stroke and Major Bleeding Among Patients With Atrial Fibrillation. J Am Heart Assoc 2016;5.
3. Zhou M, Chang HY, Segal JB, Alexander GC, Singh S. Adherence to a Novel Oral Anticoagulant Among Patients with Atrial Fibrillation. J Manag Care Spec Pharm 2015;21:1054-62.
4. Martinez C, Katholing A, Wallenhorst C, Freedman SB. Therapy persistence in newly diagnosed non-valvular atrial fibrillation treated with warfarin or NOAC. A cohort study. Thromb Haemost 2016;115:31-9.
5. Crivera C, Nelson WW, Bookhart B, Martin S, Germain G, Laliberté F, Schein J, Lefebvre P. Pharmacy quality alliance measure: adherence to non-warfarin oral anticoagulant medications. Curr Med Res Opin 2015;31:1889-95.
6. Cutler TW, Chuang A, Huynh TD, Witt RG, Branch J, Pon T, White R. A retrospective descriptive analysis of patient adherence to dabigatran at a large academic medical center. J Manag Care Spec Pharm 2014;20:1028-34.
7. Schulman S, Shortt B, Robinson M, Eikelboom JW. Adherence to anticoagulant treatment with dabigatran in a real-world setting. J Thromb Haemost 2013;11:1295-9.

13. Grouping of variables according to five WHO-Dimensions of adherence

Table 2 - Grouping of variables according to WHO dimensions of adherence

WHO-Dimension	Group of variables	included variables
**Social/economic factors**	Age	all age categories which were mentioned in the articles
	Sex	female sex
		male sex
		male gender
	Costs	cost of treatment
		costs
		high costs
		high treatment cost
		financial concerns
		concerns about costs for drugs
	Employment status	not currently working
		employment status: employed
	Deprivation index	living in more deprived municipalities (Quintile 2 vs. Quintile 1 (least deprived))
		living in more deprived municipalities (Quintile 3 vs. Quintile 1 (least deprived))
		living in more deprived municipalities (Quintile 4 vs. Quintile 1 (least deprived))
		living in more deprived municipalities (Quintile 5 vs. Quintile 1 (least deprived))
		Overseas departments vs. Quintile 1 (least deprived)
	Regional factors	*region north*
		region south
		region west
		region east
		region northeast
		region midwest
		region south west

		region west vs. South
		region northeast vs. South
		region Midwest vs. South
		living in village
		residency in Wales
		Region US-West
		Region North America
		Region Latin America
		Region Asia
	Education	higher education
**Health-care team and system-related factors**	Insurance plan	statutory health insurance
		insurance type: EPO
		insurance type: HMO
		insurance type: IND
		insurance type: POS
		insurance type: PPO
		insurance type: EPO
		insurance plan unknown vs. PPO
		insurance type: commercial vs. MAPD
	Physician characteristic and recommendation	initiation of treatment in primary care
		first prescriber is a hospital practitioner (hospital practitioner vs. GP)
		substitution with VKA by primary physician
		physician's choice
		no further need of anticoagulation
		patient at high risk when continuing DOAC
		physician's recommendation
		treating physician's decision
		substitution with VKA by primary physician
	Other	two-week limit on prescription period
**Condition-related**	Concomitant	diagnosis of anemia

**factors**	diseases/diagnosis	diagnosis of cancer
		diagnosis of cardiovascular disease
		diagnosis of dementia/cognitive impairment
		diagnosis of depression
		diagnosis of diabetes
		diagnosis of dyslipidemia
		diagnosis of heart failure
		diagnosis of hypertension
		diagnosis of ischemic heart disease (including acute coronary syndrome)
		diagnosis of left ventricular hypertrophy
		diagnosis of liver disorder
		diagnosis of liver failure/insufficiency
		diagnosis of myocardial infarction
		diagnosis of minimally symptomatic/asymptomatic AF
		diagnosis of permanent/non-permanent AF
		diagnosis of renal disorder (including worsened renal function)
		diagnosis of renal failure/insufficiency
		diagnosis of retinopathy
		diagnosis of stroke/TIA
		diagnosis of thrombocytopenia
		diagnosis of thromboembolism
		diagnosis of vascular disease
		cardiovascular risk factors
		family history of cardio-vascular risk factors
		number of concomitant diseases
		higher number of concomitant diseases
		concomitant diseases/diagnosis
		diagnosis of stroke or previous thromboembolism
		Comorbidity: total hip replacement/total knee replacement
		various medical conditions

		worsening renal function
		renal insufficiency
		diagnosis of coronary heart disease
		chronic kidney disease
		diagnosis of hyperlipidemia
		history of arterial thrombolic event
		MMSE-Score > 23
		left atrial appendage thrombus
		circulatory disorder
		thromboembolic events
		renal impairment
		diagnosis of congestive heart failure
		liver disease
		prior pulmonary embolism
		arterial hypertension
		venous thromboembolism
		major cardiovascular event
		liver impairment
		essential hypertension
		development of renal dysfunction
		development of liver dysfunction
		development of upper extremity deep venous thrombosis
		diagnosis of retinopathology
	Comorbidity index	higher Charlson-Comorbidity index
		higher comorbidity burden (higher Charlson Comorbidity Index)
		Charlson comorbidity score (1-4) vs. 0
		Charlson comorbidity score (≥5) vs. 0
		Charlson comorbidity score (≥2) vs. 0
	CHADS2-/CHA2DS2-VASc-Score	higher CHADS2 score
		higher mean CHADS2-score

		higher CHA2DS2-VASc overall score (2.43 +/-0.7 vs. 2.8 +/-0.9)
		higher CHA2DS2-VASc score
		low stroke risk (CHA2DS2VASc Score) (score of 1-2)
		CHA2DS2-VASc score = 0
		CHA2DS2-VASc score = 1
		CHA2DS2-VASc score < 2
		CHA2DS2-VASc Score=1 vs. 0
		CHA2DS2-VASc Score≥2 vs. 0
		CHADS2 < 2
		CHADS2 =0
		CHADS2 =1
		CHADS2 =2
		CHADS2 =3
		CHADS2 =4
		CHADS2 =5
		CHADS2 =6
	Bleeding risk	HEMORR2HAGES >3
		high HAS-BLED score
		perceived increased bleed risk by GP
		HAS-BLED score
	Laboratory values and clinical assessments	elevated Leucocyte count (HR per 1G/l increase)
		elevated liver enzymes
		changed lab values
		higher baseline creatinine value
		higher initial total cholesterol
		higher total cholesterol after 12 months
		higher initial cLDL
		higher cLDL after 12 months
		lower initial cHDL
		higher cHDL after 12 months

		higher initial total triglycerides
		higher total triglycerides after 12 months
		high creatinine levels (no levels indicated)
		higher initial glycemia
		higher glycemia after 12 months
		higher initial body weight
		higher body weight after 12 months
		higher height
		BMI
		higher initial abdominal waist perimeter
		higher abdominal waist perimeter after 12 months
		higher initial systolic blood pressure
		higher systolic blood pressure after 12 months
		higher initial diastolic blood pressure
		higher diastolic blood pressure after 12 months
		diagnosis of microalbuminuria
		glomerular filtration <60
		higher inital GFR
		higher GFR after 12 months
	Procedures and interventions	surgery/intervention
		GI procedures
		surgical procedures
		surgery/dentistry
		aorto-coronary bypass
		post percutaneous coronary intervention/drug eluting stent
		hemodialysis
		admission for other medical reasons
		bridging therapy start
		switching from NOAC to warfarin due to surgical procedures
		cardioversion

	Dependency/functionality	IADL
		mRS (modified Ranking Scale score) higher
		functional dependency (mRS ≥ 3) 3 months after stroke
		poor general condition
		palliative situation
		medication self-administration
		medication assisted/performed by a caregiver
		drug given by someone else
	Cure of AF	patient-reported end of AF and permanent return to sinus rhythm
		stable sinus rhythm or left atrial appendage occlusion
		stable sinus rhytm
**Therapy-related factors**	Previous experience with anticoagulation	anticoagulation-naive
		history of OAC
		previous treatment with VKA: with poor INR control (not motivated by lack of adherence or treatment)
		baseline use of oral anticoagulant
		any oral anticoagulant at baseline
		prior Dabigatran use (other dosage)
		prior Rivaroxaban use (other dosage)
		long-treatment for AF
	GI bleeding	gastrointestinal bleeding
		GIT bleeding
		haemoptysis
		major lower gastrointestinal hemorrhage
	Hematuria	haematuria
		macrohematuria
		hematuria
	Intracranial bleeding	subdural haematoma needing hospitalization and surgery
		intracranial bleeding
		intracranial hemorrhage

	Previous bleeding	previous bleeding
		previous bleeding complications
		history of bleeding
		previous bleeding, any
		prior bleeds
	Other bleeding complications	bleeding (as AE)
		bleeding
		bleeding events
		bleeding event
		clinically relevant nonmajor bleeding
		major bleeding
		major bleeding events
		major hemorrhage
		major or clinically-relevant non-major bleed
		minor bleeding
		minor bleeding
		minor bleeding events
		mucosal bleeding
		mucosal bleed
		epistaxis
		other bleeding complications
		bleeding in the mouth with loss of gingiva
		other bleeding events than intracranial and gastrointestinal
	Side effects other than bleeding	side effects
		adverse events
		serious adverse events
		other adverse events
		other non-bleeding side effects
		non-bleeding adverse event
		Adverse effects after dabigatran

		heartburn
		itching
		severe itching
		pruritus
		pruritus/rash
		skin rash
		severe pneumonia
		reflux syndrome
		dyspepsia
		gastric upset
		GI symptoms
		dyspepsia/abdominal pain
		GIT symptoms
		risk of falls
		frequent falls
		multiple falls, fractures
		fall/trauma
		hypersensitivity to agent
		hair loss
		dyspnea
		dysphagia
		bloating
		flatulence
		headache
		vertigo/nausea/fatigue
		nausea
		eczema
		diarrhea
		edema/weight loss/diarrhea
	Drug burden	higher number of drugs taken (no number indicated)

		higher number of drugs taken
		higher total daily pill burden
		higher total drug number (5.1+/-2.9 vs. 3.6+/-2.1)
		use of 5 and more additional drugs
		use of additional oral medication
		using > 3 drugs
		Comedication for other chronic and debilitating diseases
		higher number of PRN (PRN=prescribed as needed) medications prescribed per patient
		current use of platelet inhibitor
		concomitant aspirin therapy
		concomitant other antiplatelet therapy
		use of antiplatelets
		use of cardiovascular drugs
		no ACE or ARB use
		> 3 cardiovascular prescriptions in the past year
		use of antihypertensives
		current use of SSRI
		concomitant parenteral anticoagulants therapy
		concomitant PPI therapy
		no statin use
		use of lipid-lowering agents
		concomitant NSAID intake
	DOAC-specific drug properties	drug-drug-interaction
		potential drug interaction
		severe interaction with concomitant medication
		contraindication
		New contraindication for rivaroxaban
		once-daily DOAC
		twice-daily use
		dosing frequency

		≥ 2 times daily
	Prior use of different drug classes	prior PPI use
		prior H2-receptor blocker use
		previous treatment with any antithrombotic medication
		previous treatment with any cardiovascular medication
	Reduced dose of DOAC	reduced dose
		low starting dose
		use of reduced doses
		receiving reduced dose
		reduced dose
**Patient-related factors**	Drug and substance abuse	smoking
		social reason (drug, alcohol abuse)
	Health literacy	higher knowledge score
		knowledge about drug usage and disease
		not believing that drugs are needed
		not believing that "as much of drugs" as the doctor thought are needed
		lower information overload score
	Treatment satisfaction	higher treatment satisfaction score
	Patient's decisions/concerns	concerns about making mistakes when taking the drugs
		time from AF diagnosis to treatment initiation > 181 days
		non-compliance
		monitoring concerns
		forgetting to take the medication
		patient’s decision
		patient's refusal
		patients inconvenience
		patient´s wish
		patient desire
		side effects concerns
		fear of adverse events

		no antidote
		unwillingness to take medication
**NO WHO DIMENSION**	other	other/unknown/other reasons
	other	change in digestive or urinary tract after cancer surgery
	other	patient´s or physician´s preference
	other	impossibility of an adequate INR follow up (when the patient cannot access a healthcare center or home hospitalization or liaison infirmary)
	other	index date in February vs. January
		index date in May vs. January
		index date in June/July vs. January

Table 3 - Variables which are reported in the multivariate results and their included variables as reported in the original research

**Variable reported in multivariate results**	**Included variables (as reported in original research)**
Higher age	all age categories which were mentioned in the articles
Male sex	Male
	Male sex
	Female sex
Concerns about costs for drugs	Concerns about costs for drugs
Higher deprivation index	living in more deprived municipalities (Quintile 2 vs. Quintile 1 (least deprived))
	living in more deprived municipalities (Quintile 3 vs. Quintile 1 (least deprived))
	living in more deprived municipalities (Quintile 4 vs. Quintile 1 (least deprived))
	living in more deprived municipalities (Quintile 5 vs. Quintile 1 (least deprived))
Higher education level achieved	Higher education
Initiation of treatment in hospital	First prescriber is a hospital practitioner

Initiation of treatment in primary care	Initiation of treatment in primary care
Concomitant diseases/diagnosis	Comorbidity: total hip replacement/total knee replacement
Higher number of concomitant diseases	Higher number of concomitant diseases
Diagnosis of diabetes	Diagnosis of diabetes
	diabetes
Previous stroke/TIA	Previous stroke/TIA
	Prior stroke/TIA
	History of stroke or TIA
	Previous TIA or stroke
	Diagnosis of ischaemic stroke/TIA
	Ischemic heart disease
Diagnosis of ischemic heart disease	Diagnosis of ischaemic heart disease
	Diagnosis of coronary heart disease
Diagnosis of heart failure	Diagnosis of heart failure
	Diagnosis of congestive heart failure
	History of heart failure
Diagnosis of depression	Diagnosis of depression
Diagnosis of anemia	Anemia
Diagnosis of renal disorder	Chronic kidney disease
	Diagnosis of kidney disease
Diagnosis of renal failure/insufficiency	Diagnosis of chronic renal failure
	Diagnosis of hypertension
Diagnosis of hypertension	Hypertension
	History of hypertension
Diagnosis of dyslipidemia	Diagnosis of hyperlipidemia
Diagnosis of cancer	Diagnosis of cancer
Diagnosis of thromboembolism	History of arterial thromboembolic event
	Prior pulmonary embolism
Diagnosis of liver failure/insufficiency	Diagnosis of liver failure
Diagnosis of liver disorder	Liver disease
Diagnosis of minimally symptomatic/asymptomatic AF	Minimally symptomatic or asymptomatic AF
Diagnosis of permanent/non-permanent AF	Permanent AF

Diagnosis of vascular disease	History of vascular disease
	Vascular diseases
Diagnosis of dementia/cognitive impairment	Diagnosis of dementia
	MMSE-score > 23
Higher comorbidity index	higher comorbidity burden (higher Charlson Comorbidity Index)
	Higher Charlson-Comorbidity index
	Charlson comorbidity score (≥2) vs. 0
	Charlson comorbidity score (≥5)
CHA_2DS_2-VASc-Score > 0	CHA2DS2-VASc Score=1 vs. 0
	CHA2DS2-VASc Score≥2 vs. 0
	CHA2DS2VASc=0
CHADS2-/CHA2DS2-VASc-Score <2	CHADS2 < 2
	CHA2DS2-VASc score = 0
	CHA2DS2-VASc score = 1
Higher bleeding risk	HAS-BLED score
	HEMORR2HAGES >3
Higher baseline creatinine value	higher baseline creatinine value
Being underweight	normal BMI
Higher initial body weight	Higher initial body weight
Higher initial systolic blood pressure	Higher initial systolic blood pressure
Higher initial diastolic blood pressure	Higher initial diastolic blood pressure
Higher initial total cholesterol	Higher initial total cholesterol
Higher initial cLDL	Higher initial cLDL
Lower initial cHDL	Lower initial cHDL
Higher mRS (modified Ranking Scale score)	mRS (modified Ranking Scale score) higher
Drug given by somebody else	drug given by someone else
Experienced with anticoagulation	any oral anticoagulant at baseline
	History of OAC
	baseline use of oral anticoagulant
	anticoagulation-naive
Side effects	Side effects
Previous bleeding	Prior bleeds
	Previous bleeding

	Previous bleeding, any
Higher number of drugs taken	higher number of drugs taken
	use of additional oral medication
	using > 3 drugs
	use of 5 and more additional drugs
	Comedication for other chronic and debilitating diseases
Concomitant antihypertensive drugs intake	Use of antihypertensives
Concomitant lipid-lowering drugs intake	Use of lipid-lowering agents
Concomitant antiplatelet intake	Use of antiplatelets
	Concomitant aspirin therapy
	Concomitant other antiplatelet therapy
Concomitant PPI therapy	Concomitant PPI
Concomitant antidepressant drug intake	Current use of SSRI
Concomitant NSAID intake	Concomitant NSAID therapy
Concomitant parenteral anticoagulants therapy	Concomitant parenteral anticoagulants therapy
Prior PPI therapy/prior H2-receptor blocker use	Prior PPI use
	Prior H_2-receptor blocker use
Reduced dose of DOAC	Reduced dose
	Low start dose
	Receiving reduced dose
Lower information overload score	Lower information overload score
Higher drug/disease related knowledge	Higher knowledge score
	knowledge about drug usage and disease
Higher treatment satisfaction score	Higher satisfaction score
Concerns about making mistakes when taking the drugs	concerns about making mistakes when taking the drugs

14. PRISMA-P Checklist

PRISMA-P 2015 Checklist

This checklist has been adapted for use with protocol submissions to *Systematic Reviews* from Table 3 in Moher D et al: Preferred reporting items for systematic review and meta-analysis protocols (PRISMA-P) 2015 statement. *Systematic Reviews* 2015 4:1

Section/topic	#	Checklist item	Information reported		Line number(s)
			Yes	No	
**ADMINISTRATIVE INFORMATION**					
**Title**					
Identification	1a	Identify the report as a protocol of a systematic review	☒	☐	28
Update	1b	If the protocol is for an update of a previous systematic review, identify as such	☐	☒	Na
**Registration**	2	If registered, provide the name of the registry (e.g., PROSPERO) and registration number in the Abstract	☐	☒	Na
**Authors**					
Contact	3a	Provide name, institutional affiliation, and e-mail address of all protocol authors; provide physical mailing address of corresponding author	☒	☐	30
Contributions	3b	Describe contributions of protocol authors and identify the guarantor of the review	☒	☐	32-37
**Amendments**	4	If the protocol represents an amendment of a previously completed or published protocol, identify as such and list changes; otherwise, state plan for documenting important protocol amendments	☐	☒	Na
**Support**					
Sources	5a	Indicate sources of financial or other support for the review	☒	☐	40-43
Sponsor	5b	Provide name for the review funder and/or sponsor	☒	☐	40-43

Section/topic	#	Checklist item	Information reported		Line number(s)
			Yes	No	
Role of sponsor/funder	5c	Describe roles of funder(s), sponsor(s), and/or institution(s), if any, in developing the protocol	☒	☐	42-43
**INTRODUCTION**					
**Rationale**	6	Describe the rationale for the review in the context of what is already known	☒	☐	46-55
**Objectives**	7	Provide an explicit statement of the question(s) the review will address with reference to participants, interventions, comparators, and outcomes (PICO)	☒	☐	56-79
**METHODS**					
**Eligibility criteria**	8	Specify the study characteristics (e.g., PICO, study design, setting, time frame) and report characteristics (e.g., years considered, language, publication status) to be used as criteria for eligibility for the review	☒	☐	82-111
**Information sources**	9	Describe all intended information sources (e.g., electronic databases, contact with study authors, trial registers, or other grey literature sources) with planned dates of coverage	☒	☐	142-144
**Search strategy**	10	Present draft of search strategy to be used for at least one electronic database, including planned limits, such that it could be repeated	☒	☐	147-169
STUDY RECORDS					
Data management	11a	Describe the mechanism(s) that will be used to manage records and data throughout the review	☒	☐	172-193
Selection process	11b	State the process that will be used for selecting studies (e.g., two independent reviewers) through each phase of the review (i.e., screening, eligibility, and inclusion in meta-analysis)	☒	☐	172-185
Data collection process	11c	Describe planned method of extracting data from reports (e.g., piloting forms, done independently, in duplicate), any processes for obtaining and confirming data from investigators	☒	☐	193
**Data items**	12	List and define all variables for which data will be sought (e.g., PICO items, funding sources), any pre-planned data assumptions and simplifications	☒	☐	76-110
**Outcomes and prioritization**	13	List and define all outcomes for which data will be sought, including prioritization of main and additional outcomes, with rationale	☒	☐	76-110

Section/topic	#	Checklist item	Information reported		Line number(s)
			Yes	No	
**Risk of bias in individual studies**	14	Describe anticipated methods for assessing risk of bias of individual studies, including whether this will be done at the outcome or study level, or both; state how this information will be used in data synthesis	☒	☐	184-185
DATA					
**Synthesis**	15a	Describe criteria under which study data will be quantitatively synthesized	☒	☐	222-223
	15b	If data are appropriate for quantitative synthesis, describe planned summary measures, methods of handling data, and methods of combining data from studies, including any planned exploration of consistency (e.g., I^2, Kendall's tau)	☐	☒	na
	15c	Describe any proposed additional analyses (e.g., sensitivity or subgroup analyses, meta-regression)	☐	☒	na
	15d	If quantitative synthesis is not appropriate, describe the type of summary planned	☒	☐	227-248
**Meta-bias(es)**	16	Specify any planned assessment of meta-bias(es) (e.g., publication bias across studies, selective reporting within studies)	☒	☐	224-225
**Confidence in cumulative evidence**	17	Describe how the strength of the body of evidence will be assessed (e.g., GRADE)	☐	☒	na
