# Supplementary material for: The Role of Adherence Thresholds for Development and Performance Aspects of a Prediction Model for Direct Oral Anticoagulation Adherence
Source: Front Pharmacol. 2019 Feb 19;10:113. doi: 10.3389/fphar.2019.00113 (PMC6389873; doi:10.3389/fphar.2019.00113)
Supplement: Supplementary file 2 [file Data_Sheet_2.pdf]

Electronic Supplementary Material 2 to

The Role of Adherence Thresholds for Development and Performance Aspects of a Prediction Model for Direct Oral Anticoagulation Adherence

Ruff *et al.*

Frontiers in Pharmacology

Simulation protocol

We aimed to investigate how well such a prediction model would have to perform for being of clinical relevance and of practical relevance in terms of cost-effectiveness. Therefore, Modeling and Simulation (*M&S*) techniques were applied to investigate threshold effects on clinical outcomes, on prediction model performance discriminating between patients above and below these thresholds, and on its impact on the cost-effective application of such a model predicting future adherence to direct oral anticoagulants (DOAC). Supplementary Figure 1 schematically illustrates the course of action.

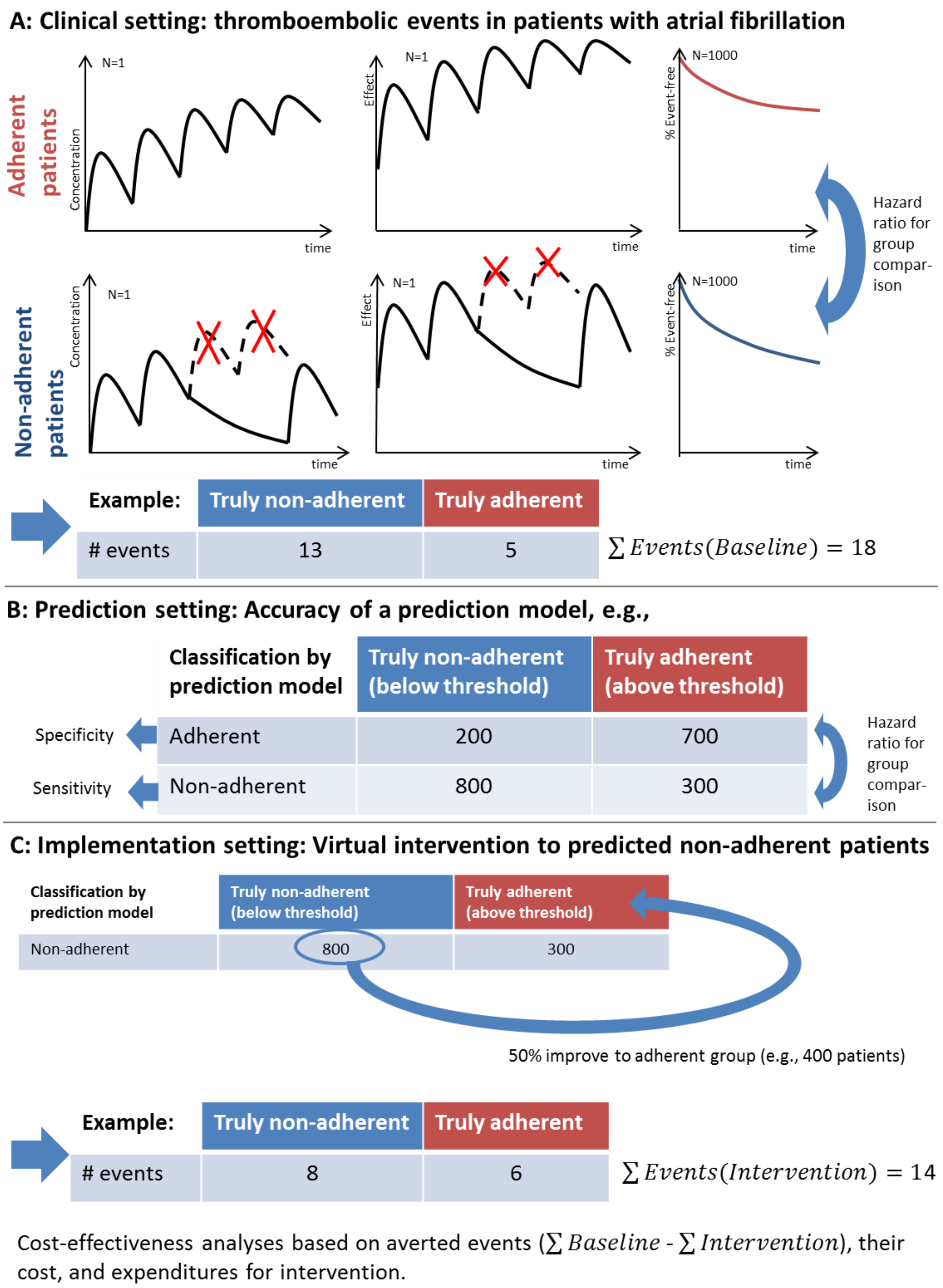

Supplementary Figure 1. Course of action combining (A) the clinical setting, (B) the prediction model setting, and (C) the implementation setting. To begin with the clinical setting (**A**), Modeling and Simulation (M&S) techniques were applied to investigate (non-)adherence and corresponding threshold effects on clinical outcomes, in particular, thromboembolic events under treatment of atrial fibrillation with the exemplary DOAC rivaroxaban. This yielded event-rates in adherence groups separated at different thresholds and the risk by comparing these groups. Concerning the prediction model setting (**B**), we examined how the risk estimate between groups below and above a certain threshold is affected by imperfect discrimination between these groups. This relates to imperfect sensitivity and specificity. If such a model would be implemented to detect patients of poor adherence (**C**), we studied the impact on the cost-effective application. Assuming that a certain proportion of poorly adhering patients below a certain threshold improves to adherence above the threshold, we calculated cost balanced by offsetting the interventional costs and saved costs by potentially averted clinical events.

Clinical setting (A): M&S of threshold-dependent adherence effects

The following investigations were based on a pharmacokinetic-pharmacodynamic (PKPD) model of rivaroxaban as an exemplary drug among the DOACs (Girgis et al., 2014). In brief, this published model linked drug intake described by a PK model to prothrombin time as an immediate PD effect. For atrial fibrillation (AF) patients with typical covariate characteristics, this model allows simulating of administration regimens and corresponding prothrombin times (PT). We chose a regimen of 20 mg rivaroxaban once daily. We derived poor adherence in terms of insufficient drug intake by randomly omitting intake times according to thresholds (e.g., 80 % adherence corresponded to a regimen with only 80 % of mandatory intakes).

For our purpose, we extended this model to incorporate a clinical effect, which we considered as the composite of stroke and systemic embolism in accordance with pivotal DOAC trials (e.g., Connolly et al., 2009 and Patel et al., 2011). We thus added an effect model in which the hazard function $h(t)$ defines the instantaneous rate of the event at time t for an individual i: $h(t) = -d/dt \cdot \log S(t)$, where $S(t)$ denotes the survival function as the probability of an event happening after some time t in the follow-up period. Within this time-to-event framework, the impact of the PD effect on the clinical outcome was implemented by an exponential survival model assuming a baseline hazard α with an effect estimate β based on the prothrombin time PT: $h(t) = \alpha \cdot e^{\beta \cdot PT}$. We adjusted these estimates to $\alpha = 0.002/24$ (i.e. on a daily scale) with an inter-individual variability of 20 % ($\omega_\alpha = 0.2$) and $\beta = -0.2$, so that simulated yearly cumulative incidences matched empiric results from pivotal DOAC trials (Connolly et al., 2009; Patel et al., 2011) and that the risk for a 10% difference in adherence approximated a hazard ratio of 1.13 (Shore et al., 2014).

Based on these assumptions, a simulation framework was set up for 1,000 virtual patients in each group above and below an adherence threshold (i.e. 2,000 in total), which were followed-up for one year. Levels of adherence were chosen in intervals of ten from 10 % to 100 % adherence. For convenience, the interval corresponding to a certain threshold was added to both groups above and below an adherence threshold. For both sides approaching the threshold, probabilities for the remaining intervals were derived from the empirical normal cumulative density function for adherence levels until the threshold, so that highest probabilities and thus most observations originated from administration regimens around the threshold. In particular, we chose four thresholds for investigation: 60 %, 70 %, 80 %, and 90 %.

Prediction setting (B): M&S of imperfect discrimination

For each threshold defining two groups above and below the respective threshold, we let a_i denote the true classification of an individual i (assigning 0 to the group above the threshold and 1 to the group below the threshold) and T_i its classification by the prediction model. Discrimination was thus defined based on conditional probabilities, so that $sensitivity = P(T_i = 1 \mid a_i = 1)$ and $specificity = P(T_i = 0 \mid a_i = 0)$. Accordingly, we re-assigned the simulated 'true' group status for various proportions of imperfect discriminations, i.e. estimates for sensitivity and specificity ranging from 0.025 to 1 in equally-spaced intervals of 0.025. This was accomplished by drawing group indicators from a Bernoulli process with response proportions ($sensitivity$ for the poorly adhering group below the threshold and $1 - specificity$ for the better adhering group above the threshold). In our simulation framework, we repeated this for 1000 draws to extract and report mean values of the simulations.

Implementation setting (C): M&S of cost savings attributed to an intervention

Focusing on these scenarios mimicking imperfect sensitivity and specificity of an intended prediction model at various adherence thresholds, a practical application of such a model would comprise an intervention targeting poor adherence. Naturally, such an intervention would have to be supplied to patients in need (i.e. with actually poor adherence) and not necessarily to well-adhering patients, where the intervention would solely take up resources. Cost-effectiveness is thus a major concern to be explored. In our *M&S* framework, we virtually offer an intervention to those patients being identified as poorly adherent (i.e. below the threshold under investigation) by an imperfectly discriminating prediction model. An intervention success rate of 50 % was assumed in all further steps; this fraction of patients with potential benefit was drawn from the subsample with good adherence (i.e. above the respective threshold). The number of events in such a re-classified data set was compared to the number of events in the original data (setting **A**). In order to study potential cost savings attributed to such a program, we defined savings as the difference between expected benefits and costs, i.e. $savings = (number\ of\ averted\ events \cdot average\ costs) - (number\ of\ interventions \cdot intervention\ costs)$. We determined the average costs for stroke and its consequences in the German health care system at 43,129 EUR (Kolominsky-Rabas et al., 2006) and set intervention cost to 100 € as a reasonable estimate within the range of possible interventions (Chapman et al., 2010)

Applied Software

Generally, time-to-event data were analyzed by the Cox proportional hazard model using the R software/environment version 3.4.0 (R Foundation for Statistical Computing, Vienna, Austria). The *mlxR* package version 3.2.4 was used for M&S steps having been cross-checked by Monolix® version 2018R1 (LIXOFT, Antony, France) for nonlinear mixed-effects modeling. Whenever applicable, statistical tests were two-tailed, 95 % confidence intervals (CI) were calculated, and P values < 0.05 were considered statistically significant.

References

Chapman RH, Ferrufino CP, Kowal SL, Classi P, Roberts CS. The cost and effectiveness of adherence-improving interventions for antihypertensive and lipid-lowering drugs*. Int J Clin Pract 2010;64:169-81.

Connolly SJ, Ezekowitz MD, Yusuf S, Eikelboom J, Oldgren J, Parekh A, Pogue J, Reilly PA, Themeles E, Varrone J, Wang S, Alings M, Xavier D, Zhu J, Diaz R, Lewis BS, Darius H, Diener HC, Joyner CD, Wallentin L. Dabigatran versus warfarin in patients with atrial fibrillation. N Engl J Med 2009;361:1139-51.

Girgis IG, Patel MR, Peters GR, Moore KT, Mahaffey KW, Nessel CC, Halperin JL, Califf RM, Fox KA, Becker RC. Population pharmacokinetics and pharmacodynamics of rivaroxaban in patients with non-valvular atrial fibrillation: results from ROCKET AF. J Clin Pharmacol 2014;54:917-27.

Kolominsky-Rabas PL, Heuschmann PU, Marschall D, Emmert M, Baltzer N, Neundörfer B, Schöffski O, Krobot KJ. Lifetime cost of ischemic stroke in Germany: results and national projections from a population-based stroke registry: the Erlangen Stroke Project. Stroke 2006;37:1179-83.

Patel MR, Mahaffey KW, Garg J, Pan G, Singer DE, Hacke W, Breithardt G, Halperin JL, Hankey GJ, Piccini JP, Becker RC, Nessel CC, Paolini JF, Berkowitz SD, Fox KA, Califf RM. Rivaroxaban versus warfarin in nonvalvular atrial fibrillation. N Engl J Med 2011;365:883-91.

Shore S, Carey EP, Turakhia MP, Jackevicius CA, Cunningham F, Pilote L, Bradley SM, Maddox TM, Grunwald GK, Barón AE, Rumsfeld JS, Varosy PD, Schneider PM, Marzec LN, Ho PM. Adherence to dabigatran therapy and longitudinal patient outcomes: insights from the veterans health administration. Am Heart J 2014;167:810-7.
